# Effect of a subset of adipose‐derived stem cells isolated with liposome magnetic beads to promote cartilage repair

**DOI:** 10.1111/jcmm.16470

**Published:** 2021-03-25

**Authors:** Aiguo Xie, Yinbo Peng, Zuochao Yao, Lin Lu, Tao Ni

**Affiliations:** ^1^ Department of Plastic and Reconstructive Surgery Shanghai Ninth People’s Hospital Shanghai Jiao Tong University School of Medicine Shanghai China

**Keywords:** adipose stem cells, cartilage, cartilage repair, chondrogenic induction, liposome magnetic bead

## Abstract

This study aimed to investigate the ability of CD146^+^ subset of ADSCs to repair cartilage defects. In this study, we prepared CD146^+^ liposome magnetic beads (CD146^+^LMB) to isolate CD146^+^ADSCs. The cells were induced for chondrogenic differentiation and verified by cartilage‐specific mRNA and protein expression. Then a mouse model of cartilage defect was constructed and treated by filling the induced cartilage cells into the damaged joint, to evaluate the function of such cells in the cartilage microenvironment. Our results demonstrated that the CD146^+^LMBs we prepared were uniform, small and highly stable, and cell experiments showed that the CD146^+^LMB has low cytotoxicity to the ADSCs. ADSCs isolated with CD146^+^LMB were all CD146^+^, CD105^+^, CD166^+^ and CD73^+^. After chondrogenic induction, the cells showed significantly increased expression of cartilage markers Sox9, collagen Ⅱ and aggrecan at protein level and significantly increased Sox9, collagen Ⅱ and aggrecan at mRNA level, and the protein expression and mRNA expression of CD146^+^ADSCs group were higher than those of ADSCs group. The CD146^+^ADSCs group showed superior tissue repair ability than the ADSCs group and blank control group in the animal experiment, as judged by gross observation, histological observation and histological scoring. The above results proved that CD146^+^LMB can successfully isolate the CD146^+^ADSCs, and after chondrogenic induction, these cells successfully promoted repair of articular cartilage defects, which may be a new direction of tissue engineering.

## 1 INTRODUCTION

Articular cartilage lacks blood supply and innervation, and it can hardly recover spontaneously after injury, so cartilage injuries caused by joint trauma, infection and degenerative diseases may cause long‐term pain, dysfunction or even disuse of the joints, bringing great suffering and inconvenience to the patients. Traditional cartilage repair methods mainly include microfracture, subchondral drilling, periosteal transplantation et al,^1^, ^2^ which aims to mobilize various cells with cartilage‐forming potential to repair and reconstruct the cartilage. However, tissue reconstructed this way is often fibrocartilage rather than hyaline, so it is likely to suffer further degradation and secondary ossification, and the overall efficacy is poor.

SUI MengHua et al^3^ took adipose tissue from goat cubs, chopped it into small pieces, digested it with type I collagenase, centrifuged and obtained a group of cells that looked like fibroblasts. After primary culture and passage, these cells appeared like bone marrow mesenchymal stem cells, with similar self‐renewal ability, long‐lasting viability and multi‐directional differentiation potential. In 2014, Li X and Gao Y et al^4^ induced differentiation of adipose stem cells into adipocytes, osteoblasts, muscle cells, cardiomyocytes, nerve cells, chondrocytes, etc with chicken amnion mesenchymal stem cells under different induction protocols, further confirming the stemness of the adipose stem cells. Acellular matrix is a natural scaffold material developed in recent years, in which the cells were removed, leaving only extracellular matrix, so it has good biocompatibility and is widely used in tissue engineering. The excellent biocompatibility of acellular matrix has been proved by in vitro and in vivo experiments that involved construction of tissue‐engineered cardiovascular, cartilage and bladder with acellular matrix.^5^, ^6^ After adequate removal of the chondrocytes, the acellular matrix basically maintained its composition and structure, which provides the material basis for cell growth and damage repair after implantation.

In this study, we isolated ADSCs by immunomagnetic separation, verified expression of cell surface antigens by flow cytometry, induced these cells into cartilage cells, verified mRNA and protein expression of cartilage markers and transplanted the induced cells into damaged joint of rats to evaluate its cartilage repair efficacy, which may provide a new treatment for articular cartilage defects.

## 2 MATERIALS AND METHODS

### 2.1 Cell lines

The ADSCs were purchased from Cyagen Bioinformatics, Inc (cat. no. HUXMD‐01001). The ADSCs were maintained to permit expansion in DMEM (Cyagen Bioinformatics, cat. no.HUXMD‐90011) with 10% FBS, 100 µ/mL penicillin and 100 mg/mL streptomycin at 37°C in 5% CO_2_ at saturated humidity. The purchased second‐generation ADSCs cells were subcultured at 80% confluence, and third‐generation cells were used for the subsequent chondrogenesis experiments.

### 2.2 Materials and instruments

Fe_3_O_4_ solution, carboxymethyl chitosan hexadecyl quaternary ammonium salt (HQCMC), DAPI were purchased from Huzhou Lieyuan Medical Laboratory Co., Ltd; BrdU, Trizol and dexamethasone reagents were purchased from Sigma; transforming growth factor β3 was purchased from Life Co., Ltd; SABC immunohistochemical staining kit and DAB kit were purchased from Boster Biological Technology co., ltd; antibodies against CD146, Sox9, collagen Ⅱ and aggrecan were purchased from CST; 1,2‐dioleoyl phosphatidylcholine (DOPC) glycidyl hexadecyl dimethylammonium chloride (GHDC), cholesterol, dichloromethane, N‐hydroxysuccinimide (NHS) and 1‐ethyl‐3‐(3‐dimethylaminopropyl) carbodiimide (EDC) were purchased from JuKang (Shanghai) Bio‐Sci & Tech Co., Ltd; BI‐90Plus laser particle size analyser/Zeta potentiometer was purchased from Brookhaven; OLYMPUS BX61 fluorescence microscope was purchased from Olympus; flow cytometer was purchased from Beckman Coulter; the electrophoresis apparatus and multi‐functional microplate reader were purchased from Bio‐Rad; the infrared imaging system was purchased from LI‐COR.

### 2.3 Preparation of CD146^+^LMB

**FIGURE 1:**
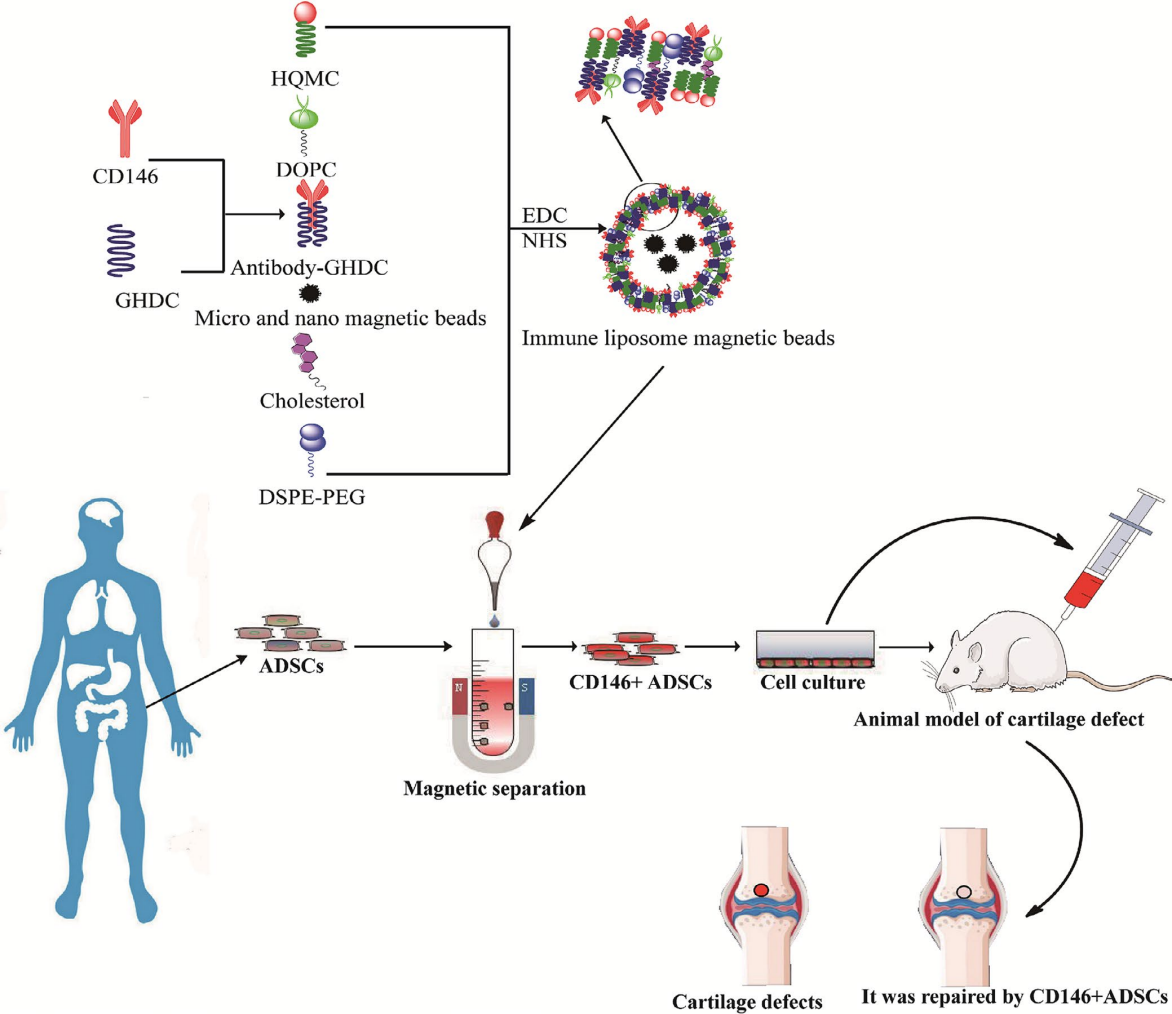
Procedure of cartilage repair using the CD146^+^LMB‐captured CD146^+^ADSCs

CD146^+^LMB were prepared by the thin‐film method. Liposomal matrix was prepared by dissolving PEG‐DSPE, cholesterol, DOPC, GHDC and HQCMC in dichloromethane, then 0.1 mol/L PBS with a pH of 7.4 was added at the same time, and the mixture was sonicated with an ultrasonic probe till it is completely emulsified to get the liposome magnetic bead (LMB) solution. 0.6 mg CD146 antibody was dissolved in 10 mL isopropanol, then N‐hydroxysuccinimide (NHS) and 1‐ethyl‐3‐(3‐dimethylaminopropyl) carbodiimide (EDC) were added separately, and the antibody solution was then mixed with the LMB solution and stirred at constant speed at 4℃ for 24 hours to get the CD146 antibody‐labelled LMB (CD146^+^LMB) (Figure 1).

### 2.4 Characterization of the CD146^+^LMB

Particle size and potential of the CD146^+^LMB were analysed by BI‐90Plus laser particle size analyser and Zeta potentiometer; morphology of the beads was observed by atomic force microscope (AFM); ultraviolet absorption spectrum of the beads was determined with a ultraviolet spectrophotometer, and magnetization of the beads was measured with a magnetic property measurement system (MPMS).

### 2.5 In vitro cytotoxicity experiments

Frozen ADSCs cells were resuscitated and cultured for 24 hours, then the medium was changed and the cells were cultured for another 24 hours. The cells were digested with trypsin, resuspended, counted with a blood cell haemocytometer and added to a 96‐well plate at 1000 cells/well in 200 μL medium, the cells were treated with 2.5, 5 or 10 μmol/L CD146^+^LMB, with DMSO as control, and cultured in the CO_2_ incubator for 24 hours; 20 μL MTT was added and incubated for 2 hours; the medium was removed and 200 μL DMSO was added to each well, and the plate was shaken gently on a horizontal shaker for 5 minutes; absorbance of each well at 560 nm was measured with a full‐wavelength microplate reader. The measurement was taken each day for 5 consecutive days with 3 duplicates, and a growth curve was plotted.

### 2.6 BrdU staining

The cells were counted using a haemocytometer and added to a 24‐well plate at 4 × 10^4^ cells/well, and the experimental group was treated with CD146^+^LMB at a final concentration of 10 μmol/L, with DMSO as control. After 72 hours of incubation, 2.5 mg/mL BrdU 2 μL was added to each well and incubated in the incubator for 40 minutes. The medium was aspirated, the cells were washed 3 times with PBS, 300 μL of 4% paraformaldehyde solution was added to each well to fix the cells at room temperature for 15 minutes, the paraformaldehyde solution was removed, the cells were washed 3 times with PBS, 1 mol/L HCl solution was added and incubated on ice for 10 minutes, and then the HCl solution was removed; 2 mol/L HCl solution was added and incubated for 5 minutes, then the cells were treated with 0.1 mol/L boric acid at room temperature for 10 minutes, washed with PBS, treated with 0.3% Triton for 20 minutes, washed with PBS again, blocked with 200 μL goat serum in each well at room temperature for 1 hours, the goat serum was removed, the cells were incubated with 200 μL anti‐BrdU antibody at 4℃ overnight, the anti‐BrdU antibody was recovered, and the cells were then incubated with immunofluorescent secondary antibody at room temperature for 2 hours; the DAPI staining solution was added and incubated in dark at room temperature for 10 minutes; the cells were washed with PBS and photographs were taken under a microscope.

### 2.7 CD146^+^ ADSCs separation and surface antigen detection

The ADSCs culture was resuspended in DMEM, counted, diluted to 1 × 10^7^ cells/mL and aliquoted into 1.5 mL sterile EP tubes at 1 mL/tube; CD146^+^LMB was washed with DMEM, added into the tubes at 10 μL/tube and incubated in at 4℃ for 30 minutes with gentle shakes every 10 minutes; then the mixture was placed on the magnetic separator for 10 minutes, the medium was removed, and the aggregated cells were washed with 1 mL aliquots of DMEM thoroughly and then transferred to 1% gelatin‐coated 6‐well plates. After another 48 hours of culture, the beads were removed and the cells were cultured for another 5 days and sorted again. Then the cells were cultured and passaged at 80%~90% confluence, and passages 2‐9 were used for subsequent experiments. During the culture, a growth curve was plotted by cell counting, and cell morphology and states were observed under a microscope with and without HE staining. Expression of surface antigens CD146, CD105, CD166, CD73, CD90, CD45, CD34 and HLA‐DR on the CD146^+^ADSCs was detected by flow cytometry.

### 2.8 Chondrogenic differentiation of the CD146^+^ADSCs

The P3 generation of ADSCs and CD146^+^ADSCs was trypsinized, and 1 × 10^6^ cells were transferred into a 15 mL sterile centrifuge tube and centrifuged at 1200 rpm for 5 minutes to form cell clusters, and then chondrogenic induction medium was added to induce differentiation. The cells were cultured in ordinary medium or induction medium (DMEM supplemented with 10% foetal bovine serum, 1 × 107 mol/L dexamethasone, 50 mol/L L‐ascorbic acid‐2‐phosphate and 10 g/L recombinant human transforming growth factor β3). The medium was refreshed every 72 hours, and cell morphology was observed with an inverted phase contrast microscope.

### 2.9 Verification of chondrogenic differentiation

Slides were taken 2 weeks after chondrogenic induction and washed with distilled water. The cells were fixed with 4% paraformaldehyde. Take the slides 2 weeks after the cartilage induction and wash them in distilled water. The cells were fixed with 4% paraformaldehyde, stained according to the instructions of AB‐PSA kit and alizarin red staining kit and observed under the microscope. Take another slide after 2‐weeks cartilage induction and wash it in distilled water. The cells were fixed with 4% paraformaldehyde and then washed with distilled water again. Add 0.2 μmol/mL chondroitinase ABC and incubate at 37°C for 30 minutes, then incubate with 3%H_2_O_2_ at room temperature for 5 minutes and wash with distilled water. Add 5%BSA at room temperature for 30 minutes and then wash with distilled water. Add Rabbit anti‐human type Ⅱ collagen polyclonal antibody diluted with 1RU 100 and incubate overnight at 4°C. According to the kit instructions, biotin‐labelled secondary antibodies were added in sequence and incubated at 37°C for 10 ~ 30 minutes. SABC and DAB colouration, haematoxylin contrast staining, anhydrous alcohol dehydration, xylene transparency, glycerol gelatin sealing are observed under a microscope. RNA was extracted from the cartilage block with Trizol, and mRNA levels of genes involved in cartilage formation: collagen Ⅱ, aggrecan and SRY‐Box 9 (Sox‐9) were determined using One‐step SYBR^®^ PrimeScript^TM^ RT‐PCR kit (TaKaRa), with GAPDH as control. The 2^‐ΔΔCt^ method was used to determine relative expression of each gene, and 3 duplicates were set for each sample. According to GenBank database, primers were designed by primerprimer6.0 software. The primers were: aggrecan: upstream CCC AAG AAT CAA GTG GAG CCG, downstream ACA CGA TGC CTT TCA CCA CGA; Collage II: upstream TGG ACG ATC AGG CGA AAC C, downstream GCT GCG GAT GCT CTC AAT CT; SOX‐9: upstream AGC GAA CGC ACA TCA AGA C, downstream CTG TAG GCG ATC TGT TGG GG; and GAPDH: upstream ATT TGG TCG TAT TGG GCG, downstream TGG AAG ATG GTG ATG GGA TT. Two weeks after chondrogenic induction, expression of chondrogenic differentiation‐related proteins: Sox9, collagen Ⅱ and aggrecan was determined by Western blot. Briefly, protein concentration was determined by the BCA method, 20 μg of the protein sample was loaded for SDS‐PAGE, and after transferring, blocking, incubating with primary and secondary antibodies, the membrane was washed and developed in the dark room. Protein bands were photographed using an infrared imaging scanner, and the grey value of the band was determined with the Quantity One software. The experiment was repeated 3 times, and the average value was taken.

### 2.10 Construction of the animal model

Under aseptic conditions, take the ADSCs and CD146^+^ADSCs after 2 weeks of cartilage induction, suspend them in 1.4% sodium alginate solution and mix them thoroughly to form a colloidal liquid for later use. The cell density is 5.0 × 10^7^/mL. Prepare a CaCl_2_ solution with a concentration of 102 mmol/L with distilled water and autoclave it for later use. Use a 200 μL pipette to suck up the mixture of sodium alginate and cells, about 20cm away from the liquid surface and drop the mixture into a CaCl_2_ solution with a concentration of 102 mmol/L to form sodium alginate microspheres that wrap the cells. Each microsphere is about 25 μL. Add chondrogenic induction medium to the 6‐well plate, put the microspheres that have been allowed to stand for 15‐20 minutes and place them in a CO_2_ saturated humidity incubator with a volume fraction of 0.05 at 37°C for 1 week.

Authors state that the use of animals in this study and this research was approved according to the relevant laws and the committee of Shanghai Ninth People's Hospital, Shanghai Jiao Tong University School of Medicine. A total of 32 BALB/C nude mice were used for the animal experiments, 2 as normal cartilage control, 10 for simple joint injury (NC), 10 for ADSCs repair after joint injury and 10 for CD146^+^ADSCs repair after joint injury. The operating environment was sterilized by UV light irradiation before operation. The nude mice were anesthetized with 0.15‐0.20 mL of 1% sodium pentobarbital (40mg/kg) via intraperitoneal injection. After anaesthesia takes effect, the nude mice are placed in lateral position and disinfected with powerful iodine in operation area. Under an 8x microscope, an arc‐shaped incision was made at the midline of the metatarsal surface of the hind limb. The incision was about 1.0 cm long, and the skin and subcutaneous tissue were incised. After sharp dissection via the subcutaneous layer toward lateral side of the knee joint, a part of the lateral femoral muscle and the lateral joint capsule were cut to get into the joint cavity. The sacrum was pushed inward, and the knee joint was bend to the flexed position to expose the articular surface of the distal femur. A 12‐gauge syringe needle (outer diameter 1.2 mm) was used to drill a hole at the flat part in the middle of the articular surface until the bevel of the needle fully entered the articular surface, causing a full‐layer articular cartilage injury with a diameter of about 1.2 mm. The ADSCs and CD146^+^ADSCs composite sodium alginate microspheres cultured by in vitro cartilage induction were made into a suitable shape and stuffed into the wound caused by the needle with a flat and normal articular surface. The wound was flushed with 0.9% physiological saline, and the lateral femoral muscle, the lateral joint capsule and the skin were sutured with the 5 “0” non‐traumatic suture needle. The mice were returned to their cages after they are fully awake.

### 2.11 Sampling and observation

The animals were killed at 12 and 24 weeks after surgery under anaesthesia, and samples of the lower femur were obtained for general observation. The samples were fixed with 10% neutral formaldehyde, decalcified with mixed decalcifying solution, dehydrated with gradient alcohol, embedded in paraffin, cut into sections, and stained with HE and toluidine blue staining to observe tissue repair. Histological scoring of the cartilage was performed on the articular cartilage semi‐quantitative scoring criteria designed by Pineda et al^7^, ^8^ The lower score shows the better repair effect.

### 2.12 Statistical analysis

SPSS21.0 statistical software was used for statistical analysis. Data are represented as mean ± standard deviation. Comparisons between time points were performed by one‐way analysis of variance; pairwise comparisons were performed by the SNK test; comparisons between two groups were performed by the *t* test. *P* <.05 was considered statistically significant (*P <.05; **P <.01; ***P <.001).

## 3 RESULTS AND ANALYSIS

### 3.1 Characterization of the CD146^+^LMB

**FIGURE 2:**
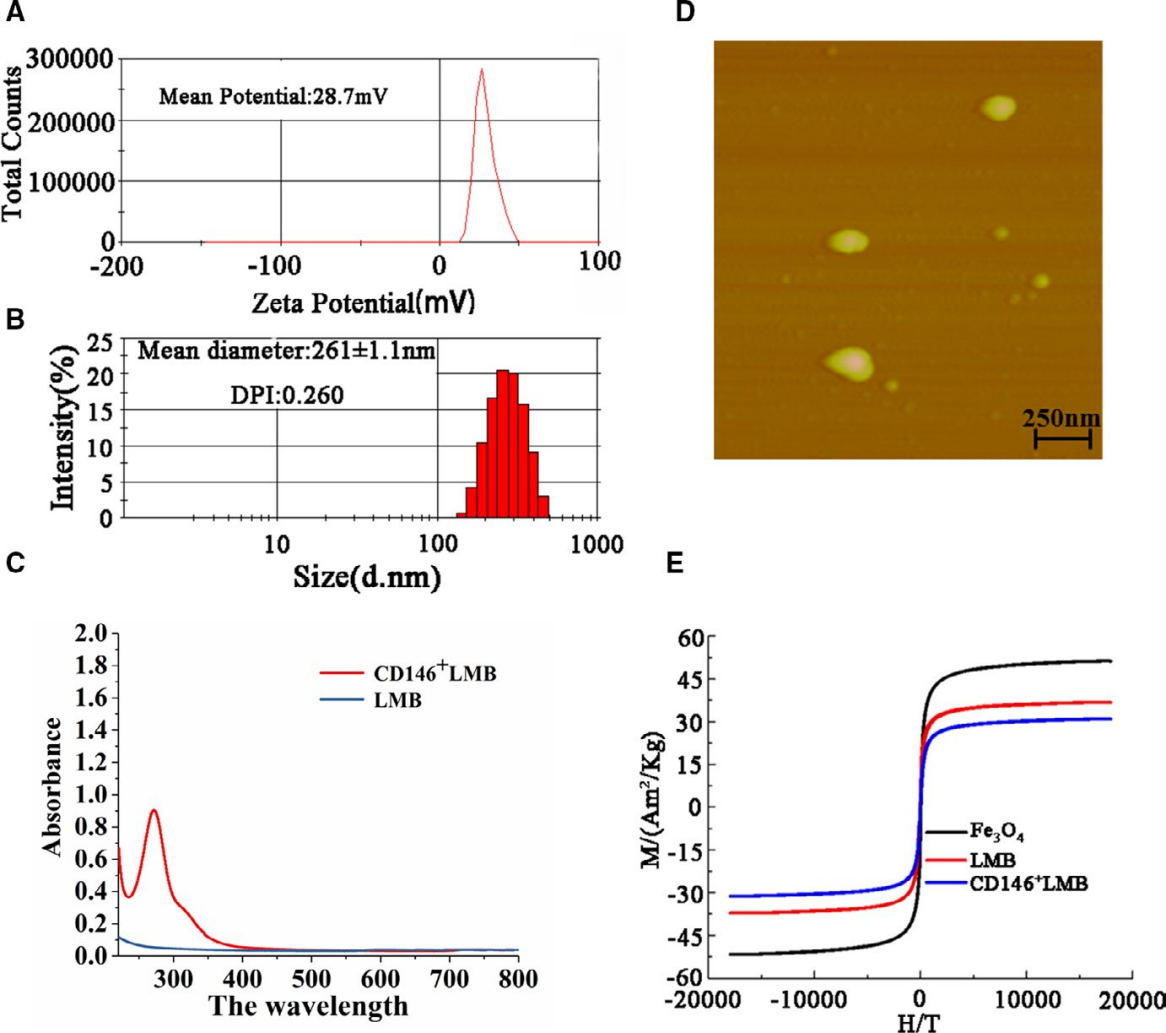
Characterization of the CD146^+^LMB: A, particle size distribution; B, potential distribution; C, ultraviolet spectrum; D, atomic force test result; E, magnetic hysteresis curve

As shown in Figure 2, the CD146^+^LMB had a + 28.7mV charge, and the charged microspheres on the surface have better dispersion due to the electrostatic repulsion between each other, which helps the beads to disperse in hydrophilic solution, and the positively charged liposome magnetic bead is easily combined with negatively charged cells^9^, ^10^ (Figure 2A); the average particle size of the beads was 261 ± 1.1 nm with a particle dispersion index (PDI) of 0.260, which indicates that the beads were satisfactorily uniform (Figure 2B); atomic force microscopy showed that the beads were microspheres in regular shape without aggregation, and the size was about 230 nm to 250 nm, in line with the result of particle size measurement. Ultraviolet spectrum test showed that CD146^+^LMB had a absorption peak at 280 nm, while LMB had no absorption peak there, indicating that protein (the anti‐CD146 antibody) had been introduced (Figure 2C); saturation magnetization of the bare magnetic sphere Fe_3_O_4_, LMB and CD146^+^LMB was 51.3Am^2^/Kg, 37.0Am^2^/Kg and 33.7 Am^2^/Kg respectively, and the decrease in saturation magnetization reflects successful DOPC encapsulation and antibody coupling.

### 3.2 Cytotoxicity of CD146^+^LMB

**FIGURE 3:**
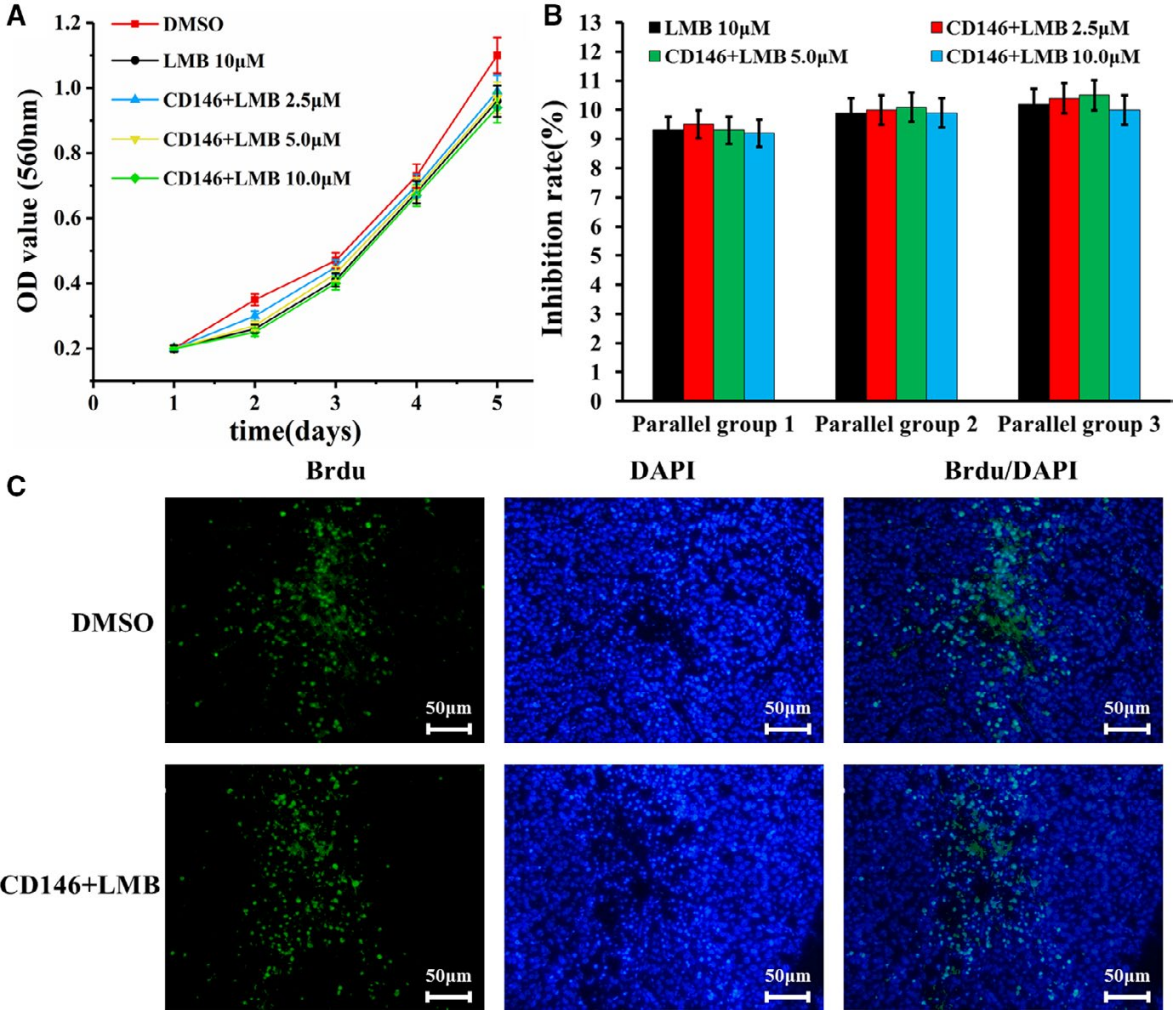
The effects of different concentrations of CD146^+^LMB on activity of the ADSCs: A, ADSCs growth curve; B, inhibition of the ADSCs by different concentrations of CD146^+^LMB. C, BrdU staining

As shown in Figure 3A, after treatment with 2.5, 5 and 10 μmol/L of CD146^+^LMB, the growth curves were not obviously different from that of DMSO control and LMB; the OD values of each group (3 duplicates for each) at Day 5 were used to calculate the inhibition rate, as shown in Figure 3B; the inhibition rate was about 7%‐9%, indicating that the CD146^+^LMB had limited cytotoxicity to the ADSCs. To further confirm our results, we stained the ADSCs with BrdU at 72 hours after treatment with 10 μmol/L CD146^+^LMB, and it was found that rate of BrdU‐positive cells was not significantly different from that of DMSO control.

### 3.3 Morphology and growth status of the CD146^+^ ADSCs

**FIGURE 4:**
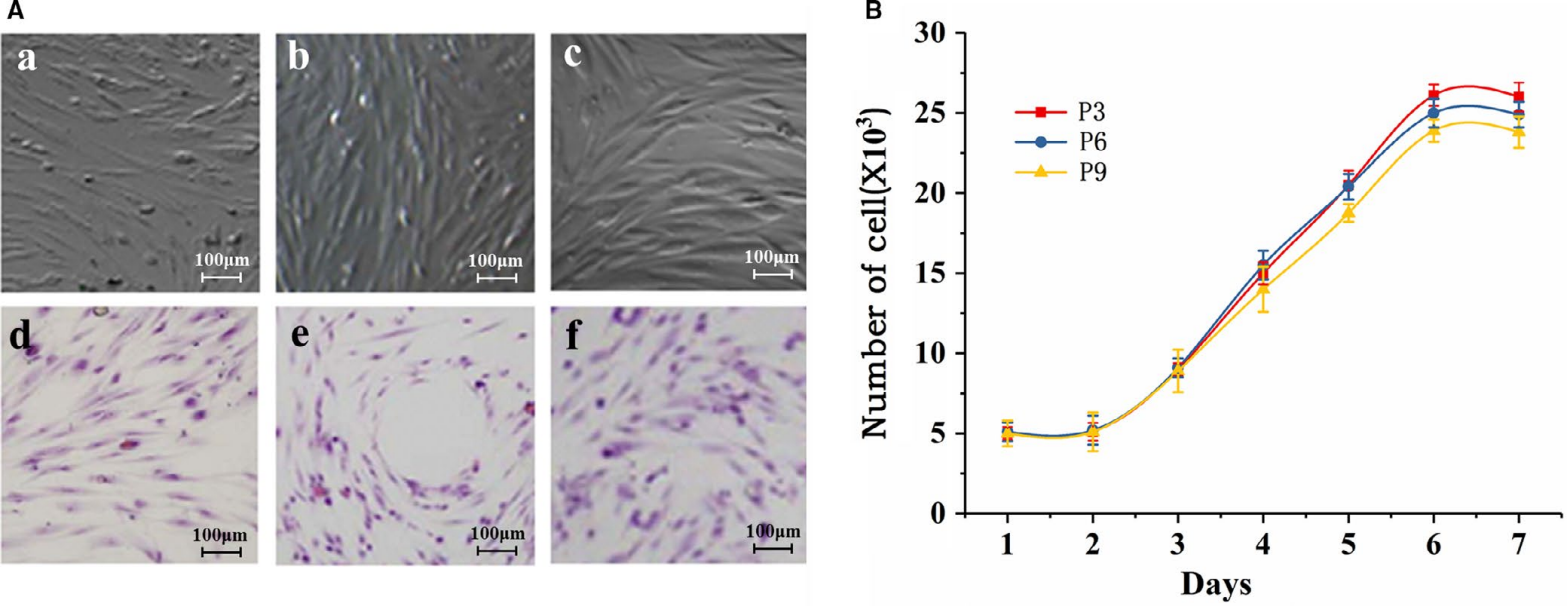
Morphology and growth status of CD146^+^ADSCs: A, cell morphology (a: P3 generation of CD146^+^ADSCs; b: P6 generation of CD146^+^ADSCs; c: P9 generation of CD146^+^ADSCs; d: HE staining of P3 generation of CD146^+^ADSCs; e: HE staining of P6 generation of CD146^+^ADSCs; f: HE staining of P9 generation of CD146^+^ADSCs; B, growth curve of the CD146^+^ADSCs

As shown in Figure 4A, most of the cells were spindle‐shaped and looked like fibroblasts and a few polygonal cells scattered. No significant differences were observed in the appearance of the P3, P6 and P9 generations of cells. Non‐adherent blood cells were removed when changing the medium, and the adherent cells grew in colonies. The cells grew to 90% of the bottom area and were arranged in order. After HE staining, the CD146^+^ADSCs had pale blue cytoplasm, were arranged in certain direction and looked like swirls of fish. It can be seen from Figure 4B that the cells were in the incubation phase in the first 2 days after inoculation and in the logarithmic growth phase for 3‐6 days, peaking on the 6th day, after which the cell growth rate slowed down and enters the plateau phase. The cell population doubled about every 60 hours.

### 3.4 Surface antigens of the CD146^+^ADSCs

**FIGURE 5:**
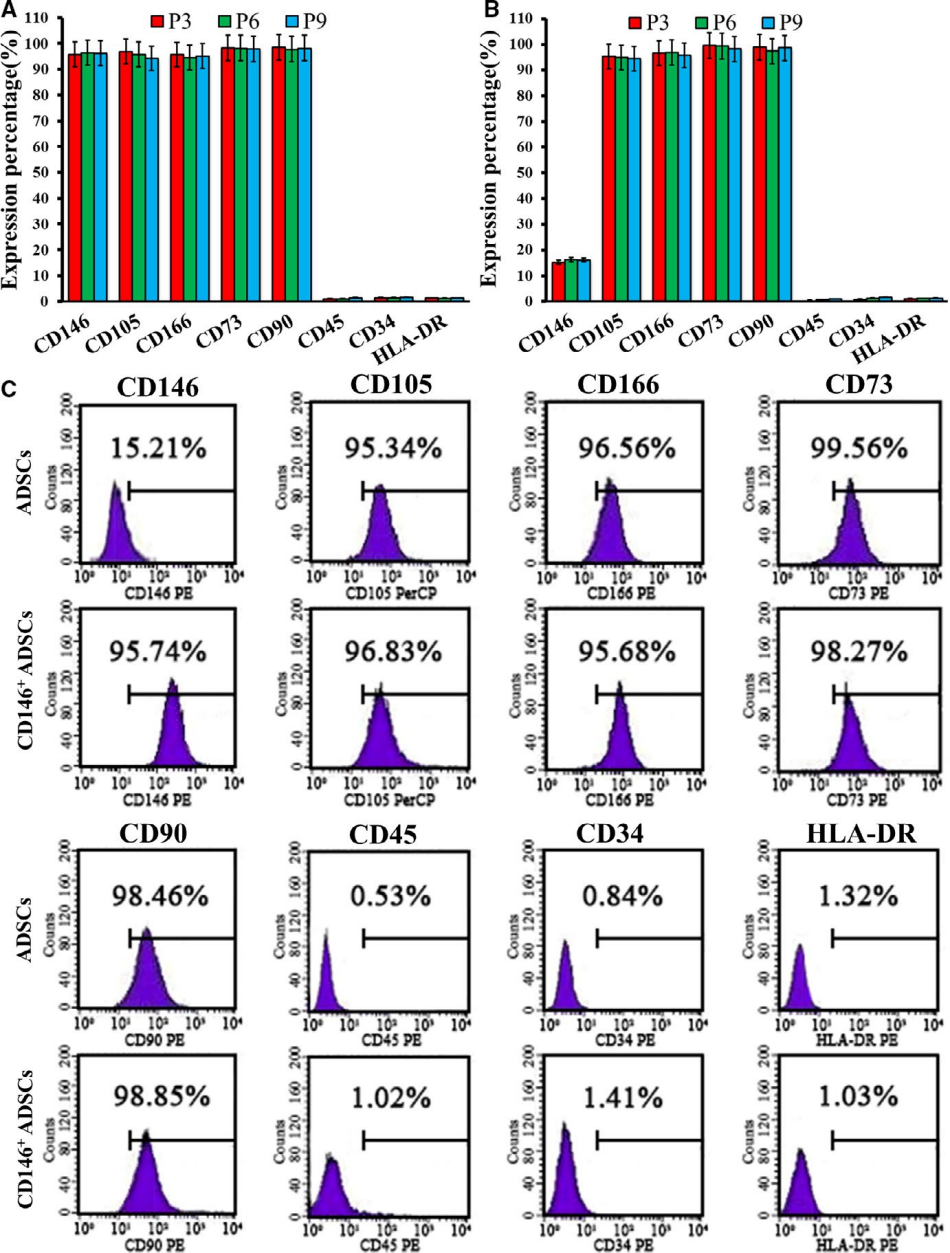
Surface antigen of the CD146^+^ADSCs: A, surface antigen of P3, P6, P9 generations of CD146^+^ADSCs; B, surface antigen of P3, P6, P9 generations of ADSCs; C, surface antigen of P3 generation of CD146^+^ADSCs

Surface antigens of P3 generation of CD146^+^ADSCs were analysed by flow cytometry, as shown in Figure 5A‐C; ADSCs cells express CD146 with positive rates of 15.21%, ADSCs cells express CD105, CD166, CD73 and CD90 with positive rates of about 95%, but do not express CD34, CD45 and HLA‐DR, and CD146^+^ADSCs cells express CD146, CD105, CD166, CD73 and CD90 with positive rates of about 95%, but do not express CD34, CD45 and HLA‐DR, which is similar to the surface antigen profile of bone marrow mesenchymal stem cells. Further analysis of P3, P6 and P9 generations of the CD146^+^ADSCs suggested that the surface antigen profile was stable.

### 3.5 Verification of chondrogenic differentiation

**FIGURE 6:**
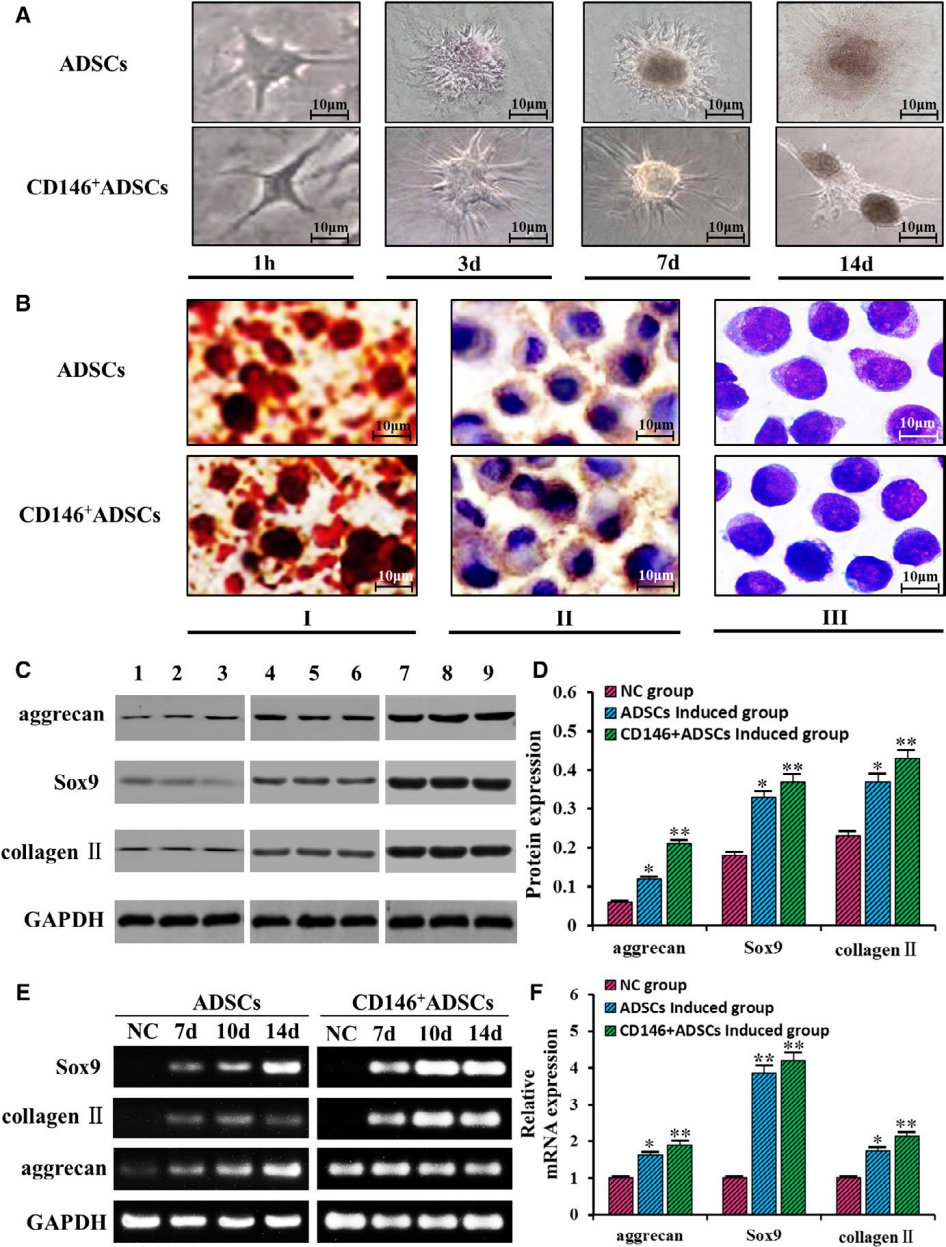
In vitro chondrogenic induction results: A, morphological changes of ADSCs and CD146^+^ADSCs; B, staining results of the ADSCs and CD146^+^ADSCs after chondrogenic induction (I: alizarin red staining of the chondrogenic induction group; II: collagen Ⅱ staining of the chondrogenic induction group; III: AB‐PSA staining of the chondrogenic induction group); C, Western blot to detect protein expression of the chondrocyte markers (1‐3 control group, 4‐6 ADSCs chondrogenic induction group, 7‐9 CD146^+^ADSCs chondrogenic induction group); D, relative expression of related proteins; E, RT‐PCR to detect mRNA expression of the chondrocyte markers; F, expression of related mRNAs by the ADSCs and CD146^+^ADSCs after chondrogenic induction

Early after chondrogenic induction, in ADSCs and CD146^+^ADSCs, no obvious changes in cell morphology were observed; 2‐3 days later, ADSCs and CD146^+^ADSCs cells at sites of higher density gradually changed from the long spindle shape similar to that of fibroblasts to short spindle or polygonal shapes. As the induction proceeded, cells at sites of higher density gradually aggregate and stacked up. In the centre of colony, a ridge or crater shape is formed, where the cells tend to be spherical, while the surrounding cells were spindle‐shaped and spread out. ADSCs after 7 days of induction, a dark yellow substance could be seen on the surface of the aggregated cells, the extracellular matrix gradually accumulated, wrapping the cell mass, and the process peaked at Day 14 after induction. CD146^+^ADSCs after 7 days of induction, a yellowish substance could be seen on the surface of the aggregated cells, the extracellular matrix gradually accumulated, wrapping the cell mass, and the process peaked at Day 14 after induction (Figure 6A). After 3 weeks of induction, the ADSCs‐ and CD146^+^ADSCs‐derived chondrocytes were stained with AB‐PSA, immunohistochemistry against collagen Ⅱ and alizarin red. As shown in Figure 6B, ADSCs were stained purple‐red by AB‐PSA with a light blue centre, and CD146^+^ADSCs were stained purple‐red by AB‐PSA with a dark blue centre; ADSCs and CD146 + ADSCs collagen Ⅱ staining was positive on cell surface and intercellular components; ADSCs alizarin red was weak positive, and CD146 + ADSCs alizarin red was positive. These results indicate obvious bone formation, and the osteogenic effect of CD146^+^ADSCs is significantly better than that of ADSCs.

After 2 weeks of induction, total RNA was extracted and analysed by RT‐PCR. It was found that mRNA levels of collagen Ⅱ, Sox9 and aggrecan significantly increased in the ADSCs and CD146^+^ADSCs induction group compared to the control group, and the mRNA expression of CD146^+^ADSCs group was increased than that of ADSCs group (Figure 6F) (*P* <.05). And as shown in Figure 6E, mRNA levels of collagen Ⅱ and Sox 9 significantly increased after 7 days of induction (*P* <.05), but aggrecan was not significantly increased at this time (*P* >.05). In cartilage formation at Day 10 and 14, expression of all 3 mRNAs significantly increased compared to Day 0 (*P* <.05 for all), and Western blot demonstrated that protein levels of Sox9, collagen Ⅱ and aggrecan were all significantly increased in the ADSCs and CD146^+^ADSCs chondrogenic induction group than the control group (0.33 ± 0.06, 0.37 ± 0.03 and 0.12 ± 0.03 of the ADSCs induction group, and 0.37 ± 0.04, 0.43 ± 0.05 and 0.21 ± 0.03 of the CD146^+^ADSCs induction group vs 0.18 ± 0.06, 0.23 ± 0.02 and 0.06 ± 0.02 of the control group) (*P* <.05 for all) as shown in Figure 6B and Figure 6C.

### 3.6 Histological observation and scoring

**FIGURE 7:**
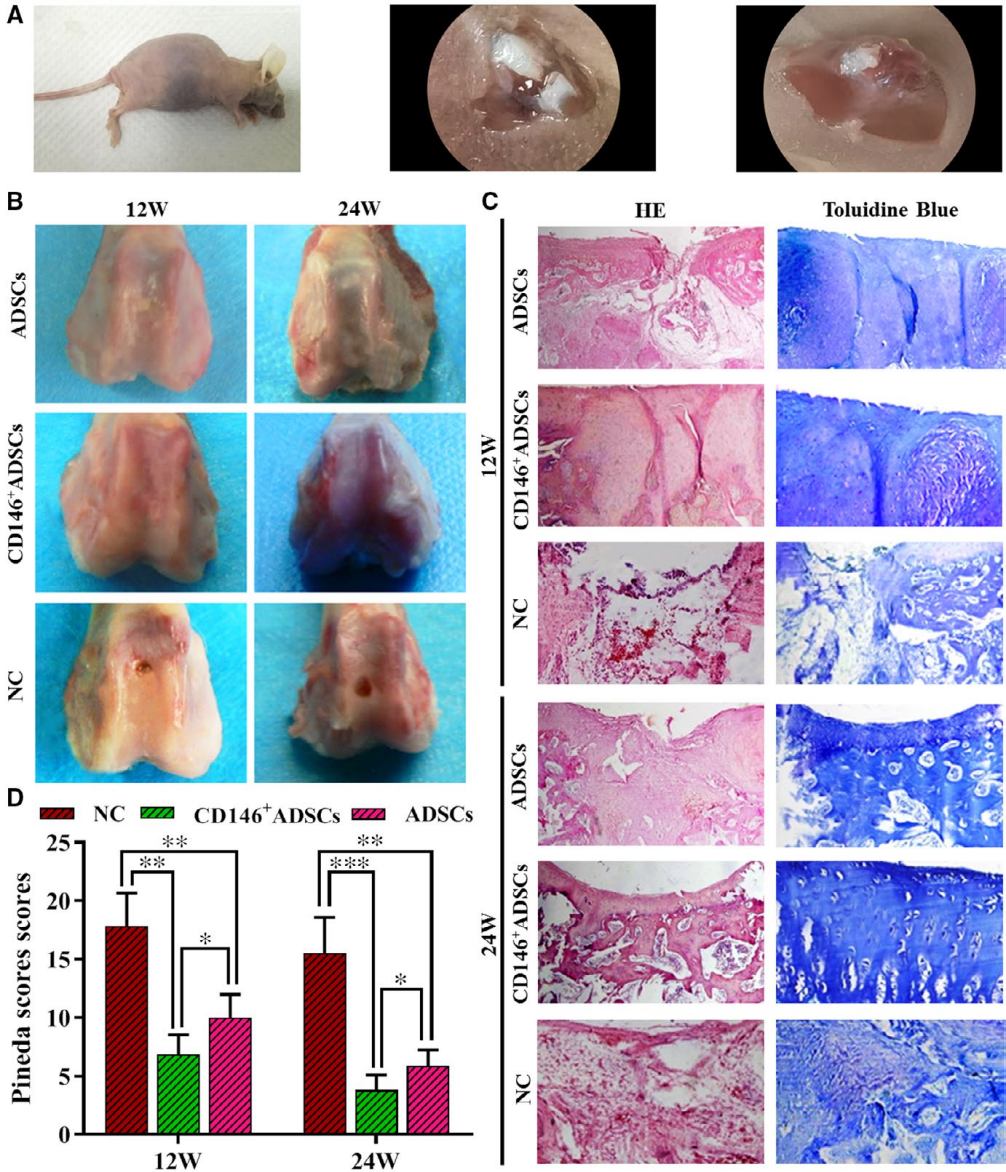
Histological observation and scoring results: A, cartilage damage model; B, repair of the cartilage defect; C, HE and toluidine blue staining of the repaired cartilage; D, histological score of the repaired cartilage

The cartilage defect model was constructed as shown in Figure 7A and was observed at 12 and 24 weeks after construction. As shown in Figure 7B, at 12 weeks, the cartilage defect in the ADSCs group and CD146^+^ADSCs group was filled with white semi‐translucent membrane‐like tissue, similar to normal cartilage in colour, the cartilage surface was relatively flat without obvious local dent, the regenerated cartilage was tightly connected to the surrounding normal cartilage, the boundaries were unclear, it is hard to touch, and CD146^+^ADSCs group is more obvious than ADSCs group; cartilage surface of the blank control (NC) group had obvious defect, was markedly dented and filled with a small amount of white membranous tissue; at 24 weeks, the cartilage defect in the ADSCs group and CD146^+^ADSCs group was filled with white semi‐translucent cartilage‐like tissue, similar to normal cartilage in colour and hardness, the cartilage surface was flat, the regenerated cartilage was tightly connected to the surrounding normal cartilage and the boundaries were disappeared, same hardness as normal cartilage, and CD146^+^ADSCs group is more obvious than ADSCs group; cartilage defect of the blank control (NC) group was covered with white membranous tissue, and there is obvious dent and a clear boundary between the defect and surrounding normal cartilage. As shown in Figure 7C, HE and toluidine blue staining at 12 weeks showed that the ADSCs group and CD146^+^ADSCs group had a flat surface and varied in thickness, the regenerated chondrocyte clusters had a distinct matrix staining, the scaffold material is equivalent to the subchondral bone area has been partially filled with new cancellous bone trabecula, the tide mark's has a tendency to recover, most of the scaffold material has degraded, and CD146^+^ADSCs group is more obvious than ADSCs group; the blank control (NC) group had a small amount of fibrous tissue in the defect, the surrounding cartilage was partially degraded, and the matrix was not stained; at 24 weeks, the ADSCs group and CD146^+^ADSCs group showed obvious matrix staining and the tide line largely recovered, morphology and structure of the subchondral bone as well as thickness of the regenerated cartilage were all close to normal, the regenerated cartilage was well integrated with surrounding normal cartilage, the surface was smooth and flat, surface cartilage cells were basically parallel with the articular surface, and CD146^+^ADSCs group is more obvious than ADSCs group; the blank control (NC) group had the defect filled with a surface layer of fibrous tissue, the matrix was not stained, and an obvious dent was observed. Histological scoring was also performed at 12 and 24 weeks; the results showed that the ADSCs group and CD146^+^ADSCs group had lower score than the blank control (NC) group (*P* <.05) and that the CD146^+^ADSCs group had lower score than the ADSCs group (*P* <.05) (Figure 7D).

## 4 DISCUSSION

ADSCs has low immunogenicity^11^ and is highly adaptive to its environment, which provide great prospects for future clinical applications.^12^, ^13^ Martinez‐Gonzalez et al^14^ confirmed that ADSCs can protect the alveolar structure of asthmatic patients by reducing neutrophil‐related inflammation, reducing IgE secretion and inhibiting lymphocyte infiltration. In addition, Won et al^15^ found that injection of ADSCs can promote hair growth. After proper induction, ADSCs can differentiate into fat, bone, cartilage, muscle and neural precursor cells.^16^, ^17^, ^18^ Due to the poor regenerative capability, tissue engineering has become a promising way to repair cartilage defects tissue transplantation technology. So far, various types of materials such as biopolymer‐based hydro‐gels,^19^ synthetic polymer‐derived scaffolds or hydrogels,^20^, ^21^ alginate^22^, ^23^, ^24^ and inorganic particle‐based nanocomposite hydro‐gels^25^ have been employed for cartilage regeneration. Among them, alginate have been widely applied as attractive materials owing to their biocompatibility, biodegrade ability, bioactivity, and cost‐effectiveness. Alginic acid is a natural polysaccharide copolymer of β‐D‐mannuronic acid and α‐L‐guluronic acid, which has been extensively studied for its biomedical application. Sodium alginate can form a gel by crosslinking with ionic or covalent bonds under mild conditions, which enables its widespread use in tissue engineering, cell encapsulation and other applications.^22^, ^23^, ^24^, ^26^ As a surface marker of vascular pericytes and smooth muscle cells, CD146 plays an important role in cell adhesion, embryonic development, immune response, angiogenesis and cancer.^27^ Studies in vitro and in vivo have both proved that the perivascular cells of CD146^+^, as a part of MSCs, play an important role in the process of wound healing.^28^ CD146^+^ADSCs promotes wound healing by secreting high levels of cytokines, such as vascular endothelial growth factor (VEGF), heparin‐binding epidermal growth factor (HB‐EGF) and basic fibroblast growth factor (bFGF).^29^ Therefore, it is of great significance to sort and culture the CD146^+^ADSCs subgroups of ADSCs cells for tissue repair. In this study, CD146^+^LMB isolation system was established for the first time to separate CD146^+^ADSCs subsets, composite hydrogels were formed by mixing sodium alginate and CaCl_2_ at various blending ratios, followed by crosslinking with calcium ions, and the isolated CD146^+^ADSCs subsets were used to induce cartilage in vitro. CD146^+^ADSCs in promoting the repair of articular cartilage injury was systematically studied by using ADSCs as a control, and the function of CD146^+^ADSCs subsets was clarified, and finally the role of CD146^+^ADSCs subsets in promoting cartilage repair was determined.

Compared with other adult stem cells, ADSCs have a wide range of sources, can be easily obtained and grow stably and rapidly in culture. For these reasons, more and more researchers hope to use it as the seed cells to build tissue‐engineered cartilage.

Chondrogenic differentiation of ADSCs is a very complicated process, many factors are involved, and the molecular mechanism has not been clarified. As we now know, SOX‐9 transfection, treatment with cytokines such as TGF‐β, appropriate oxygen concentration, matrix encapsulation and continuous stress can all induce cartilage phenotype from mesenchymal stem cells. In addition, many experiments have shown that cell aggregation in vitro is the prelude to cartilage formation.^30^, ^31^ In this study, it was also observed that with rapid growth of the ADSCs, the intercellular space became smaller and disappeared, and the cells aggregated and stacked up where the cell density was high. After inducing chondrogenesis, cell aggregation was more obvious, and ridges were formed. Aggregation of cells occurred before formation of extracellular matrix, which was consistent with the natural process of early cartilage formation. As the ADSCs differentiated toward chondrocytes, the cartilage‐specific matrix components were expressed, and the expression levels increased with time. Among them, the most important is Sox9, which regulates chondrogenic differentiation. Molecular biology techniques have demonstrated the importance of this gene for cartilage formation. Sox9 is a major transcription factors involved in chondrogenic differentiation during embryogenesis, while RUNX2 is a major regulator of osteogenesis. Zhou et al^32^ revealed using mouse and human cells that Sox9 is a major regulator of differentiation: when it is expressed in stem cells, it shuts down RUNX2, leading to chondrogenic differentiation, and the expression of Sox9 and collagen Ⅱ is closely related. In addition, Sox9 can also mediate the expression of insulin‐like growth factor 1 and interleukin‐1 which also affect chondrocyte synthesis and catabolism.^33^, ^34^ Bi et al^35^ found through experiments that Sox9‐deficient chondrocytes did not express type II, IX, XI collagens and aggrecan, confirming Sox9 as a key gene in the process of chondrocyte differentiation and cartilage formation. On the other hand, Tsuchiya^36^ et al transfected stem cells with a vector carrying the Sox9 gene and found that they can induce cartilage phenotype from stem cells.

In this study, after chondrogenic induction, the ADSCs isolated with CD146^+^LMB expressed Sox9, collagen Ⅱ, and aggrecan, as shown by RT‐PCR and Western blot, suggesting that the CD146^+^ADSCs had the potential to differentiate into chondrocytes, which was in line with the definition of stem cells by the International Cell Transplantation Society,^37^ indicating that such cells have the characteristics of osteochondral precursor cells. When passaged to the third generation, the stemness of these cells were stable, indicating that the adipose‐derived chondrocyte precursor cells may become ideal seed cells for cartilage tissue engineering. Sox9 expression, positive AB‐PSA staining, collagen Ⅱ staining, and alizarin red staining results all indicated chondrogenic differentiation of the ADSCs. In recent years, with the continuous development of tissue engineering, the application of stem cells in cartilage tissue engineering has received more and more attention. Studies have shown that under appropriate conditions, stem cells can not only be induced to differentiate into cartilage in vitro, but also continue to form cartilage and assist repair of joint cartilage defects in vivo.^38^, ^39^, ^40^ In this study, gross observation, histological observation and histological scoring of the animal model all showed that the ability of the CD146^+^ADSCs‐derived chondrocytes to repair cartilage was significant compared to the blank control group, the CD146^+^ADSCs‐derived chondrocytes to repair cartilage was significant compared to the ADSCs group, and the regenerated cartilage was close to normal.

In summary, the CD146^+^LMB prepared in this study successfully isolated CD146^+^ADSCs, and when implanted in animal models after chondrogenic induction, it can effectively repair the defect of cartilage and subchondral bone in 24 weeks; the effective repair is better than ADSCs. This study demonstrated the feasibility of isolating specific subsets of ADSCs with immunomagnetic bead method and showed the function of a subset of CD146^+^ADSCs in tissue repair, which may be a new direction of tissue engineering.

## CONFLICT OF INTEREST

The authors declare that they have no competing interests.

## AUTHOR CONTRIBUTIONS

**Aiguo Xie:** Methodology (equal); Writing‐original draft (equal); Writing‐review & editing (equal). **Yinbo Peng:** Conceptualization (equal); Writing‐original draft (equal); Writing‐review & editing (equal). **Zuochao Yao:** Data curation (lead); Formal analysis (lead); Investigation (lead); Writing‐review & editing (supporting). **Lin Lu:** Project administration (equal); Resources (equal); Supervision (equal); Validation (equal); Writing‐review & editing (supporting). **Tao Ni:** Project administration (equal); Resources (equal); Supervision (equal); Validation (lead); Writing‐review & editing (supporting).

## Data Availability

The data that support the findings of this study are available from the corresponding author upon reasonable request.
